# Cost effectiveness of therapist delivered cognitive behavioural therapy and web-based self-management in irritable bowel syndrome: the ACTIB randomised trial

**DOI:** 10.1186/s12876-021-01848-9

**Published:** 2021-07-06

**Authors:** Paul McCrone, Hazel Everitt, Sabine Landau, Paul Little, Felicity L. Bishop, Gilly O’Reilly, Alice Sibelli, Rachel Holland, Stephanie Hughes, Sula Windgassen, Kim Goldsmith, Nicholas Coleman, Robert Logan, Trudie Chalder, Rona Moss-Morris

**Affiliations:** 1 Health Services and Population Research Department, Institute of Psychiatry, Psychology and Neuroscience, King’s College London, London, UK; 2 Primary Care and Population Sciences, University of Southampton, Southampton, UK; 3 Department of Biostatistics and Health Informatics, Institute of Psychiatry, Psychology and Neuroscience, King’s College London, London, UK; 4 Department of Psychology, University of Southampton, Southampton, UK; 5 Department of Psychology, Institute of Psychiatry, Psychology and Neuroscience, King’s College London, London, UK; 6 Department of Gastroenterology, University Hospital Southampton, Southampton, UK; 7 Department of Gastroenterology, King’s College Hospital, London, London, UK; 8 Department of Psychological Medicine, Institute of Psychiatry, Psychology and Neuroscience, King’s College London, London, UK; 9 Institute for Lifecourse Development, University of Greenwich, London, UK

**Keywords:** Irritable bowel syndrome, Cognitive behavioural therapy, Economic evaluation, Cost-effectiveness

## Abstract

**Background:**

Telephone therapist delivered CBT (TCBT) and web-based CBT (WCBT) have been shown to be significantly more clinically effective than treatment as usual (TAU) at reducing IBS symptom severity and impact at 12 months in adults with refractory IBS. In this paper we assess the cost-effectiveness of the interventions.

**Methods:**

Participants were recruited from 74 general practices and three gastroenterology centres in England. Interventions costs were calculated, and other service use and lost employment measured and costed for one-year post randomisation. Quality-adjusted life years (QALYs) were combined with costs to determine cost-effectiveness of TCBT and WCBT compared to TAU.

**Results:**

TCBT cost £956 more than TAU (95% CI, £601–£1435) and generated 0.0429 more QALYs. WCBT cost £224 more than TAU (95% CI, − £11 to £448) and produced 0.029 more QALYs. Compared to TAU, TCBT had an incremental cost per QALY of £22,284 while the figure for WCBT was £7724. After multiple imputation these ratios increased to £27,436 and £17,388 respectively. Including lost employment and informal care, TCBT had costs that were on average £866 lower than TAU (95% CI, − £1133 to £2957), and WCBT had costs that were £1028 lower than TAU (95% CI, − £448 to £2580).

**Conclusions:**

TCBT and WCBT resulted in more QALYs and higher costs than TAU. Complete case analysis suggests both therapies are cost-effective from a healthcare perspective. Imputation for missing data reduces cost-effectiveness but WCTB remained cost-effective. If the reduced societal costs are included both interventions are likely to be more cost-effective.

*Trial registration* ISRCTN44427879 (registered 18.11.13).

## Background

Irritable bowel syndrome (IBS) is a common condition with a prevalence of 10–20% [1]. Because of clinical symptoms including abdominal pain, altered bowel habit, and bloating, IBS has a negative impact on quality of life of those experiencing it [2]. Treatment options for IBS are varied, and include pharmacological interventions, life style changes, and psychological therapies [3]. Given the limited availability of healthcare resources it is imperative to assess the relative cost-effectiveness of alternative ways of providing care.

Economic evaluations of treatments for IBS have been conducted previously. A study from Sweden compared internet therapy with usual care [4]. At 12-month follow-up the intervention group had lower severity of symptoms and lower societal costs, and a 79% likelihood of being a dominant option. Previously, McCrone et al. found that nurse-delivered CBT in addition to mebeverine had reasonable likelihood of cost-effectiveness after three months but not beyond this [5]. Elsewhere, acupuncture has been evaluated in a trial as a treatment for IBS compared to usual care [6]. Quality-adjusted life years (QALYs) were higher for the acupuncture group but costs were higher, resulting in an incremental cost-effectiveness ratio of £62,500 which exceeds the threshold of £20,000 to £30,000 used in England by the National Institute for Health and Care Excellence (NICE).

We recently conducted a three-arm randomised controlled trial (n = 558) comparing telephone-delivered cognitive behavioural therapy (TCBT), web-delivered cognitive behavioural therapy (WCBT) and treatment as usual (TAU) [7, 8]. Both TCBT and WCBT were superior to TAU in terms of improvements in the primary outcomes, IBS symptom severity and impact of IBS on life roles (daily functioning) up to 12 months follow-up. They were also superior on a range of secondary outcomes (global symptom relief, anxiety, depression, and cognition). The aims of this paper are to (1) investigate the differences in health service and societal costs between participants allocated to TCBT, WCBT or TAU and (2) compare cost-effectiveness of TCBT and WCBT compared to TAU in terms of quality-adjusted life years (QALYs) over the 12-month follow-up.

## Methods

### Setting and sample

The study recruited from primary care (74 general practices) and secondary care (three gastroenterology centres) settings in the UK between May 2014 and March 2016. The sample consisted of adults with refractory IBS (clinically significant symptoms for 12 months despite being offered first line therapies). Full details of the inclusion and exclusion criteria and flow through the trial are provided in the published protocol and clinical results [7, 8].

### Interventions

Participants were randomised with an independent web-based service at the level of the individual with varying block sizes to receive TCBT plus TAU, WCBT plus TAU or TAU alone. TCBT consisted of a patient CBT self-management manual [9], six 60-min telephone sessions over nine weeks, and two 60-min booster sessions at four and eight months (eight hours of therapist time). Although therapist training and supervision was provided, we have assumed that the cost of this are contained within the unit cost used for therapist time. WCBT consisted of interactive, tailored web-based CBT (WCBT), three 30-min telephone sessions over nine weeks, and two 30-min boosters at four and eight months (two and a half hours of therapist time). TAU consisted of usual healthcare consultations and medication. Those delivering therapy could not be blinded to treatment. The economic analyses required knowledge if which therapy was provided and so economists were also not blinded.

### Outcomes

Baseline assessments were conducted with follow-ups at three, six, and 12 months following randomisation. The two co-primary outcome measures were the IBS Symptom Severity Score (IBS SSS) and the Work and Social Adjustment Scale (WSAS). The IBS SSS is a 500 point scale with higher scores indicating greater symptomatology [10], while the WSAS is a 40 point scale with higher scores indicating lower levels of daily functioning [11]. The main outcome measure used in the health economic analyses was the EQ-5D-5L [12]. This instrument scores five domains (mobility, self-care, usual activities, pain/discomfort, and anxiety/depression) as 1 (representing no problems), through to 5 (extreme problems). The resulting five-digit score represents a unique health state and this is converted to a value anchored by 1 (full health) and 0 (death). Area under the curve methods are then used to combine these weights at baseline and each follow-up to generate QALYs. This method assumed a linear change between quality of life scores [13].

### Service use and costs

Costs were measured from both a health service and a societal perspective (the latter including family care costs and lost employment costs). TCBT and WCBT costs were based on the number of telephone sessions with therapists and the unit cost of therapist time. This was calculated using a therapy cost of £98 per session reported in the Personal Social Services Research Unit’s annual compendium [14], adjusted for the time spent using therapy. The median length of TCBT sessions was 55 min and combined with the £98 this produced a cost per minute of therapist time which was combined with TCBT and WCBT therapy time. The development and maintenance costs (including the fee for hosting the site) of WCBT were estimated and apportioned appropriately. This was apportioned over trial participants and was estimated at £13.51 per participant in the trial. (Clearly if the intervention were rolled out to a wider population then the development costs per person would become very small.) Use of other services was measured with the self-report Client Service Receipt Inventory (CSRI) at baseline (for the previous six months) and each follow-up (covering the period since the previous interview) [15]. Services included in the CSRI primary and secondary healthcare contacts, inpatient stays, investigations, and medication. Costs were generated by combining this information with appropriate unit cost information for 2015/16 [14, 16, 17]. These service costs were added to the intervention costs. The unit costs used are available in the Appendix.

Costs to society were estimated by including informal care costs and those resulting from time off work. Informal care costs were calculated by asking participants to say how much time per week family members and friends typically spent providing help in specific areas due to problems associated with IBS. This time was combined with an average hourly wage rate of £15.73 to represent the opportunity cost of informal care [18]. The number of days lost from work was recorded and combined with an average daily wage rate of £105 to generate lost production costs. We did not include patient time costs incurred while using the interventions.

### Analyses

Comparisons of costs were made at three, six and 12 months as well as the entire follow-up period, with baseline costs controlled for. (This is common practice in health economic studies given the usual wide variation in costs. Such variation can often be associated with baseline costs.) The costs of the interventions were only added to the costs for the whole follow-up period. The cost comparisons used regression models with baseline costs entered as an independent variable along with site variables as stratifiers and with bootstrapped percentile 95% confidence intervals generated around the coefficients. Medication costs were calculated but excluded from the analyses due to substantial missing data.

Cost-effectiveness analyses were conducted from a healthcare perspective on cases where data on both costs and QALYs were available. Incremental cost-effectiveness ratios were produced, and uncertainty addressed using cost-effectiveness planes (CEPs) and cost-effectiveness acceptability curves (CEACs). The CEPs and CEACs were produced from incremental costs and QALYs generated from bootstrapped resamples of the original data. The range of threshold values used in the CEAC was £0 to £60,000 (so including the £20,000-£30,000, which guides decisions made by the National Institute for Health and Care Excellence in England).

We conducted sensitivity analyses by changing the intervention costs upwards and downwards by 25% and 50%. This was done because in other settings therapy might be delivered by professionals at a higher or lower pay grade. Sensitivity analyses were also conducted by using the minimum wage of £7.50 per hour to calculate informal care and lost days from work (assuming 7.5 h per day) and by using the unit cost of a home care worker (£20 per hour) to value informal care. Furthermore, missing cost and EQ-5D-5L data were imputed using multiple imputation methods on 1000 bootstrapped resamples. We assumed that data were missing at random and that potential predictors of costs and EQ-5D-5L scores (at each follow-up point) were corresponding costs and EQ-5D-5L scores from other time points and also the primary clinical measures (IBS-SSS and WSAS). The imputation was conducted on each bootstrapped resample in turn and used chained equations and predictive mean matching based on the five nearest neighbours. The mean of the cost and QALY differences from the 1000 resamples was extracted and reported.

Patient and public involvement (PPI) representatives participated in the trial management group and trial steering committee and were included in all phases of trial design, including planning recruitment and recruitment materials.

## Results

The sample at baseline numbered 558 and is described in detail elsewhere with a consort diagram of patient flow for clinical outcomes [8]. Baseline clinical and demographic characteristics are shown in Table 1 and these indicate that at baseline the groups were well balanced. Around three-quarters of the participants were female and nearly all of white ethnicity. Only about one-tenth had seen a specialist for IBS symptoms even though the duration of symptoms was on average over six years. Psychiatric comorbidity, as indicated by cut-offs on the HADS scores, was relatively high, particularly for anxiety.

**Table 1:** Demographic and clinical characteristics of the study sample at baseline

		TCBT N = 186	WCBT N = 185	TAU N = 187	All N = 558
Age (years)	Mean (SD)	43·4 (12·5)	43·8 (13·6)	42·0 (13·5)	43·1 (13·2)
Sex	Female (%)	139 (74·7)	145 (78·4)	139 (74·3)	423 (75·8)
Ethnicity (%)	White	162 (87·1)	171 (92·4)	174 (93·0)	507 (90·9)
Deprivation score (IMD 2010)	Mean (SD)	16·7 (12·0)	17·3 (12·3)	17·2 (11·8)	17·1 (12·0)
Seen a GI consultant for IBS	Count (%)	20 (10·7%)	15 (8·1%)	22 (11·8%)	57 (10·2%)
Years of symptoms before diagnosis	N/median	186/3·29	185/3·0	187/3·0	558/ 3·0
Duration of IBS (years)	N/median range	185/6·5 1·0–65·0	185/8·6 0·7–45·4	187/6·3 0·3–49·9	557/7·4 0·3–64·6
HADS anxiety score	Mean (SD) case* N/(%)	10·6 (4·3) 89/186 (47·9%)	11·1 (4·3) 98/185 (53·0%)	10·5 (4·0) 96/187 (51·3%)	10·7 (4·2) 283/558 (50·7%)
HADS depression Score	Mean (SD) case* N/(%)	5·5 (3·6) 47/186 (25·3%)	5·9 (3·8) 60/185 (32·4%)	5·6 (3·5) 50/187 (26·7%)	5·7 (3·7) 157/558 (28·1%)
IBS subtype IBS Diarrhoea	No. (%)	60 (32.3)	60 (32.4)	58 (31.0)	178 (31.9)
IBS constipation	No. (%)	26 (14.0)	23 (12.4)	27 (14.5)	76 (13.6)
IBS alternating	No. (%)	93 (50.3)	98 (53.0)	96 (51.3)	287 (51.5)
IBS unclassified	No. (%)	6 (3.2)	4 (2.2)	6 (3.2)	16 (2.9)
Unknown	No. (%)	60 (32.3)	60 (32.4)	58 (31.0)	178 (31.9)

*HADS case of anxiety defined as scoring $\geqq$ 10/21 and depression as scoring $\geqq$ 7/21 on subscale

### Service use

Service use and lost employment data were available for 186 TCBT, 185 WCBT and 187 TAU participants at baseline. At three-month follow-up the figures were 142 (76% of baseline number) for TCBT, 132 (71%) WCBT and 134 (72%) TAU; six-month follow-up 135 (73%) TCBT, 115 (62%) WCBT and 128 (68%) TAU; 12-month follow-up 130 (70%) TCBT, 120 (65%) WCBT and 130 (70%) TAU. During the six-month period prior to baseline assessment, over four-fifths of participants had contacts with a GP (Table 2). The use of other doctors, practice nurses and pharmacists were relatively high compared to use of other services. Few participants had inpatient stays. More than half of each group used medication related to IBS. For about three-quarters of the sample, investigations (usually blood tests) were performed. Around one-fifth of each group received care from family or friends because of health problems. In the period up to three-month follow-up, about half the participants in each group had GP contacts. Pharmacists contacts was the next most used service. There were only slight differences between groups in terms of use of services. In the next period, up to the six-month follow-up, there were similar levels of service use as observed previously. Around half the participants received GP care and around one-quarter had contacts with pharmacists. In the final six-month period, up to 12-month follow-up, the most commonly used services were GPs, other doctors, pharmacists, and practice nurses. Use of inpatient care was slightly higher than previously.

**Table 2:** Number (%) of participants using services at baseline and each follow-up by treatment group

	Baseline			0–3 month follow-up			3–6 month follow-up			6–12 month-follow-up		
	TCBT (n = 186)	WCBT (n = 185)	TAU (n = 187)	TCBT (n = 142)	WCBT (n = 132)	TAU (n = 134)	TCBT (n = 135)	WCBT (n = 115)	TAU (n = 128)	TCBT (n = 130)	WCBT (n = 120)	TAU (n = 130)
Gastroenterologist	29 (16)	28 (15)	37 (20)	6 (4)	5 (4)	12 (9)	4 (3)	2 (2)	10 (8)	7 (5)	8 (7)	15 (12)
GP	149 (80)	160 (86)	162 (87)	71 (50)	67 (51)	69 (51)	71 (53)	52 (45)	68 (53)	82 (63)	71 (59)	73 (56)
Other doctor	37 (20)	38 (21)	51 (27)	18 (13)	20 (15)	21 (16)	19 (14)	16 (14)	20 (16)	42 (32)	27 (23)	28 (22)
Pharmacist	53 (28)	60 (32)	67 (36)	41 (29)	37 (28)	32 (24)	35 (26)	25 (22)	32 (25)	44 (34)	34 (28)	32 (25)
Physiotherapist	18 (10)	19 (10)	24 (13)	14 (10)	9 (7)	15 (11)	19 (14)	8 (7)	14 (11)	16 (12)	12 (10)	12 (9)
Practice nurse	67 (36)	61 (33)	80 (43)	23 (16)	27 (20)	29 (22)	23 (17)	27 (23)	27 (21)	27 (21)	26 (22)	25 (19)
Home nurse	1 (1)	0 (0)	0 (0)	0 (0)	0 (0)	0 (0)	0 (0)	0 (0)	0 (0)	1 (1)	0 (0)	2 (2)
Hospital nurse	11 (6)	14 (8)	21 (11)	3 (2)	14 (11)	6 (4)	6 (4)	5 (4)	7 (5)	13 (10)	11 (9)	11 (8)
Psychiatrist	5 (3)	6 (3)	0 (0)	2 (1)	0 (0)	1 (1)	0 (0)	1 (1)	2 (2)	2 (2)	1 (1)	0 (0)
Social worker	4 (2)	2 (1)	2 (1)	1 (1)	2 (2)	1 (1)	2 (1)	2 (2)	1 (1)	3 (2)	1 (1)	1 (1)
Other therapist	15 (8)	21 (11)	16 (9)	7 (5)	11 (8)	7 (5)	5 (4)	9 (8)	6 (5)	7 (5)	7 (6)	6 (5)
Acupuncturist	10 (6)	5 (3)	11 (6)	6 (4)	1 (1)	3 (2)	5 (4)	2 (2)	4 (3)	8 (6)	1 (1)	6 (5)
Dietician	27 (15)	23 (12)	29 (16)	4 (3)	3 (2)	7 (5)	0 (0)	3 (3)	9 (7)	2 (2)	6 (5)	5 (4)
Homeopath	8 (4)	5 (3)	4 (2)	3 (2)	0 (0)	3 (2)	3 (2)	0 (0)	0 (0)	4 (3)	2 (2)	2 (2)
OT	3 (2)	3 (2)	1 (1)	1 (1)	1 (1)	1 (1)	3 (2)	0 (0)	0 (0)	0 (0)	2 (2)	1 (1)
Osteopath	12 (6)	15 (8)	12 (6)	7 (5)	6 (5)	8 (6)	9 (7)	8 (7)	7 (5)	7 (5)	5 (4)	8 (6)
Inpatient	13 (7)	11 (6)	18 (10)	3 (2)	2 (2)	2 (1)	3 (2)	1 (1)	3 (2)	8 (6)	5 (4)	7 (5)
A & E	24 (13)	19 (10)	27 (14)	7 (5)	8 (6)	9 (7)	8 (6)	9 (8)	7 (5)	14 (11)	7 (6)	15 (12)
Medication	99 (53)	109 (59)	108 (58)	50 (35)	56 (42)	64 (48)	45 (33)	39 (34)	58 (45)	48 (37)	46 (38)	60 (46)
Investigations	147 (79)	138 (75)	151 (81)	45 (32)	42 (32)	42 (31)	44 (33)	24 (21)	35 (27)	62 (48)	42 (35)	51 (39)
Informal care	36 (19)	36 (19)	37 (20)	21 (15)	20 (15)	26 (19)	24 (18)	19 (17)	21 (16)	13 (10)	15 (13)	22 (17)
Lost work days	96 (52)	74 (40)	91 (49)	33 (23)	36 (27)	45 (34)	39 (29)	42 (37)	47 (37)	48 (37)	52 (43)	61 (47)

GP, general practitioner; OT, occupational therapis, A & E accident and emergency department

Table 3 shows the mean number of service contacts for those participants who had at least one contact. For most services the number of is less than ten and there are few notable differences between the groups. However, for those who received informal care, the TAU group had more hours a week than the other two groups during the follow-up periods.

**Table 3:** Mean number of service contacts among participants with at least one contact at baseline and each follow-up by treatment group

	Baseline			0–3 month follow-up			3–6 month follow-up			6–12 month-follow-up		
	TCBT (n = 186)	WCBT (n = 185)	TAU (n = 187)	TCBT (n = 142)	WCBT (n = 132)	TAU (n = 134)	TCBT (n = 135)	WCBT (n = 115)	TAU (n = 128)	TCBT (n = 130)	WCBT (n = 120)	TAU (n = 130)
Gastroenterologist	1·5	2·8	2·6	1·2	1·4	1·2	2·3	11·0	1·3	1·3	1·6	3·7
GP	3·9	3·8	4·2	2·4	2·1	3·0	2·0	2·3	2·7	2·0	2·4	2·6
Other doctor	3·6	2·1	3·4	2·3	1·8	1·8	4·7	2·1	1·5	1·6	2·1	2·6
Pharmacist	3·6	4·4	4·4	3·0	2·7	2·8	2·0	2·2	2·0	2·0	2·1	2·6
Physiotherapist	3·2	6·5	6·0	3·4	3·0	1·9	3·6	2·9	3·7	3·9	4·3	3·2
Practice nurse	2·1	2·2	1·7	1·7	1·6	1·3	1·9	1·3	1·5	1·6	1·5	1·6
Home nurse	2·0	–	–	–	–	–	–	–	–	20·0	–	2·0
Hospital nurse	2·7	2·0	1·4	1·7	1·7	1·2	2·2	1·6	1·3	1·8	1·4	1·8
Psychiatrist	3·0	2·0	–	2·5	–	1·0	–	3·0	2·0	2·5	2·0	–
Social worker	3·5	6·0	1·0	3·0	6·0	1·0	3·0	1·0	1·0	2·3	1·0	4·0
Other therapist	5·7	7·3	5·9	5·6	3·8	3·9	4·4	4·8	2·0	2·9	5·0	2·7
Acupuncturist	4·4	4·8	5·4	1·8	5·0	2·0	3·6	2·0	1·5	4·1	1·0	3·3
Dietician	1·7	1·6	1·8	1·0	3·0	1·4	–	1·0	1·7	1·0	2·2	1·4
Homeopath	2·5	2·4	11·0	3·3	–	1·0	1·7	–	–	1·0	1·0	2·5
OT	4·3	3·3	1·0	6·0	3·0	1·0	1·7	–	–	–	4·0	1·0
Osteopath	3·1	4·0	3·3	1·7	2·3	2·4	2·3	1·3	1·7	1·9	5·2	2·1
Inpatient	–	–	–	–	–	–	–	–	–	–	–	–
A & E	1·3	1·2	1·3	1·0	1·1	1·6	1·0	1·3	1·0	1·1	1·0	1·3
Medication	–	–	–	–	–	–	–	–	–	–	–	–
Investigations	2·6	2·7	2·6	1·8	2·5	2·0	1·9	1·6	1·9	3·1	2·0	2·0
Informal care	9·6	11·5	12·9	6·7	7·5	10·7	7·2	4·8	26·0	5·6	10·0	16·5
Lost work days	10·3	10·2	9·8	8·5	5·1	5·3	9·9	3·1	6·9	7·9	4·3	11·5

GP, general practitioner; OT, occupational therapis, A & E accident and emergency department

### Costs

At baseline, inpatient care, although used by few participants, had the highest costs, along with GP and contacts with other doctors (Table 4). The groups did not differ much in terms of costs of particular services. Total healthcare costs were on average £681 for TCBT, £620 for WCBT, and £802 for TAU. TAU participants had costs that were £122 more than for TCBT and £182 more than for WCBT. With informal care and lost employment costs included, the total was £1995 for TCBT, £1965 for WCBT, and £2352 for TAU. TCBT had total costs that were £357 less than for TAU and WCBT had total costs that were £387 less than for TAU.

**Table 4:** Mean cost of service contacts at baseline and each follow-up by treatment group (2015/16 £s)

	Baseline			0–3 month follow-up			3–6 month follow-up			6–12 month-follow-up		
	TCBT (n = 186)	WCBT (n = 185)	TAU (n = 187)	TCBT (n = 142)	WCBT (n = 132)	TAU (n = 134)	TCBT (n = 135)	WCBT (n = 115)	TAU (n = 128)	TCBT (n = 130)	WCBT (n = 120)	TAU (n = 130)
Gastroenterologist	32	57	69	7	7	14	9	26	14	9	15	59
GP	106	110	122	39	36	50	34	35	47	42	47	48
Other doctor	97	57	124	39	36	37	90	39	32	70	65	77
Pharmacist	26	36	40	22	19	17	13	12	13	17	15	16
Physiotherapist	15	33	38	16	10	11	25	10	20	24	21	14
Practice nurse	11	11	11	4	5	4	5	5	5	5	5	5
Home nurse	< 1	0	0	0	0	0	0	0	0	4	0	1
Hospital nurse	7	7	7	2	8	2	4	3	3	8	5	7
Psychiatrist	11	9	0	5	0	1	0	4	4	5	2	0
Social worker	3	3	< 1	1	4	< 1	2	1	< 1	2	< 1	1
Other therapist	36	66	40	22	25	16	13	30	7	12	23	10
Acupuncturist	12	6	16	4	2	2	7	2	2	13	< 1	8
Dietician	20	16	22	2	6	6	0	2	9	1	9	4
Homeopath	5	3	12	4	0	1	2	0	0	2	1	2
OT	6	4	< 1	3	2	1	3	0	0	0	5	1
Osteopath	10	16	10	4	5	7	8	4	5	5	11	7
Inpatient	163	71	156	56	121	13	24	17	17	202	36	103
A & E	23	16	27	7	9	14	8	14	8	17	8	20
Medication	23	28	36	6	12	15	5	6	12	11	15	20
Investigations	98	99	108	35	52	32	36	22	21	81	55	43
Total health cost	681	620	802	271	346	227	280	224	206	519	325	393
Informal care	759	915	1048	203	232	423	261	161	873	230	510	1144
Lost work days	556	430	502	208	145	186	299	118	265	307	195	566
Total cost	1995	1965	2352	682	723	836	840	503	1344	1055	1029	2103

GP, general practitioner; OT, occupational therapis, A & E accident and emergency department

In the period up to three-month follow-up the costs again were relatively high for GP and other doctor contacts. Inpatient costs for the WCBT group were higher than for the other groups. The average healthcare costs excluding the intervention were £271 for TCBT, £346 for WCBT, and £227 for TAU. Adjusting for baseline, the TCBT group had healthcare costs that were £54 higher than for TAU (bootstrapped 95% CI, − £61 to £174) and WCBT had costs that were £151 higher than for TAU (bootstrapped 95% CI, − £71 to £510). Mean societal costs (including informal care and lost employment) were £682 for TCBT, £723 for WCBT, and £836 for TAU. Adjusting for baseline, TCBT had mean total costs that were £25 higher than for TAU (bootstrapped 95% CI, − £321 to £386) and WCBT had mean total costs that were £218 higher than for TAU (bootstrapped 95% CI, − £236 to £665).

In the three months up to the six-month follow-up, costs were highest for GPs and other doctors. Average non-intervention healthcare costs were £281 for TCBT, £224 for WCBT, and £206 for TAU. After adjusting for baseline costs TCBT had £86 higher costs than TAU (bootstrapped 95% CI, − £33 to £234) and WCBT had costs that were £39 higher than for TAU (bootstrapped 95% CI, − £68 to £155. Informal care costs were very different between groups during this period. Average societal costs were £840 for TCBT, £503 for WCBT, and £1344 for TAU. Adjusting for baseline resulted in TCBT having costs that were on average £343 less than for TAU (bootstrapped 95% CI, − £266 to £1022) and WCBT having costs that were £420 lower than for TAU (bootstrapped 95% CI, − £57 to £1009).

In the six months up to the 12-month follow-up, average healthcare costs (excluding the intervention) were £519 for TCBT, £325 for WCBT, and £393 for TAU. After adjusting for baseline, TCBT had non-intervention healthcare costs that were on average £144 more than for TAU (bootstrapped 95% CI, − £119 to £499) and WCBT had costs that were £42 lower than for TAU (bootstrapped 95% CI, − £124 to £210). The higher level of informal care costs for TAU continued during this period. The TAU group also had higher lost employment costs than the other groups. Mean societal costs (including informal care and lost employment) were £1055 for TCBT, £1029 for WCBT, and £2103 for TAU. After adjusting for baseline, TCBT had costs that were £873 lower than for TAU (bootstrapped 95% CI, − £223 to £2153) and WCBT had costs that were £751 lower than for TAU (bootstrapped 95% CI, − £180 to £1885).

Mean (SD) total healthcare costs, with ACTIB CBT costs included, over the whole one-year follow-up period were £1650 (£1931) for TCBT, £943 (£955) for WCBT, and £715 (£884) for TAU. Adjusting for baseline, TCBT had costs that were on average £943 higher than for TAU which was statistically significant (bootstrapped 95% CI, £572 to £1363) and WCBT had costs that were £278 higher than for TAU which was also significant (bootstrapped 95% CI, £11 to £514). Cost differences for participants where EQ-5D-5L data were available (which is relevant for the complete case analysis) were £956 (bootstrapped 95% CI, £601 to £1435) for TCBT and £224 for WCBT (bootstrapped 95% CI, − £11 to £448).

The mean (SD) societal costs over the one-year follow-up period, again including ACTIB CBT costs, were £3065 (£5179) for TCBT, £2094 (£3069) for WCBT, and £4374 (£11,843) for TAU. After adjusting for baseline, TCBT had costs that were £866 lower than TAU (bootstrapped 95% CI, − £1133 to £2957) and WCBT had costs that were £1028 lower than for TAU (bootstrapped 95% CI, − £448 to £2580).

### Quality-adjusted life years

Response to the EQ-5D-5L was relatively low (Table 5). Utility scores were high for each group at each time point. Improvements were greater for the TCBT and WCBT groups compared to TAU. After adjustment for baseline EQ-5D-5L utility scores, it was shown that TCBT resulted in 0.0414 more QALYs than TAU (95% CI, 0.0194 to 0.0635) and WCBT resulted in 0.0269 more QALYs than TAU (95% CI, 0.0041 to 0.0497). Differences in QALYs for those with follow-up cost data were 0.0429 (95% CI, 0.0205 to 0.0653 and 0.0290 (95% CI, 0.0063 to 0.0518) respectively. Table 5 also reveals that there was a substantial amount of missing EQ-5D-5L data. Reasons for this are addressed in the Discussion.

**Table 5:** EQ-5D-5L utility scores by time period and quality-adjusted life years (QALYs)

	TCBT (n = 186)		WCBT (n = 185)		TAU (n = 187)	
	N (%)	Mean (sd)	N (%)	Mean (sd)	N (%)	Mean (sd)
Baseline	185 (99)	0·8191 (0·1283)	185 (100)	0·8016 (0·1651)	187 (100)	0·8101 (0·1468)
0–3 month follow-up	147 (79)	0·8499 (0·1253)	133 (72)	0·8392 (0·1657)	132 (71)	0·8083 (0·1547)
3–6 month follow-up	134 (72)	0·8761 (0·1128)	112 (60)	0·8563 (0·1404)	128 (68)	0·8251 (0·1438)
6–12 month follow-up	120 (65)	0·8799 (0·1425)	113 (61)	0·8459 (0·1513)	123 (66)	0·8265 (0·1497)
QALYs	106 (57)	0·8786 (0·0786)	92 (50)	0·8525 (0·1244)	102 (55)	0·8254 (0·1313)

### Cost-effectiveness results—complete case analyses

Data were available on healthcare costs and QALYs for 99 (53%) TCBT participants, 92 (50%) WBT participants, and 100 (53%) TAU participants. Dividing incremental healthcare costs by incremental QALYs differences for those with both cost and QALY data results in the following incremental cost-effectiveness ratios (ICERs): TCBT v TAU £22,284 and WCBT v TAU £7724. The ICER for TCBT compared to WCBT was £52,662.

**Fig. 1:**
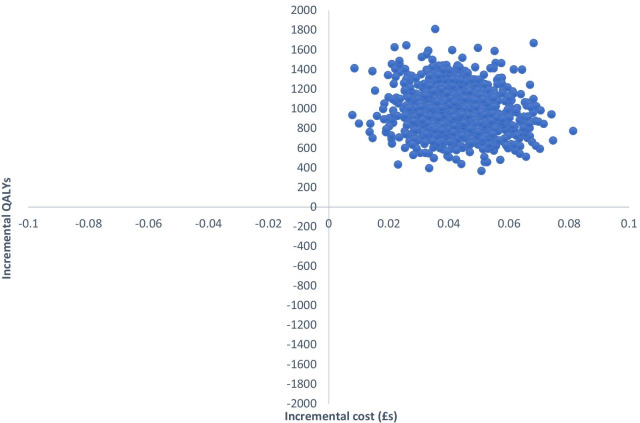
Cost-effectiveness plane for TCBT versus TAU (healthcare perspective, complete case analysis)

**Fig. 2:**
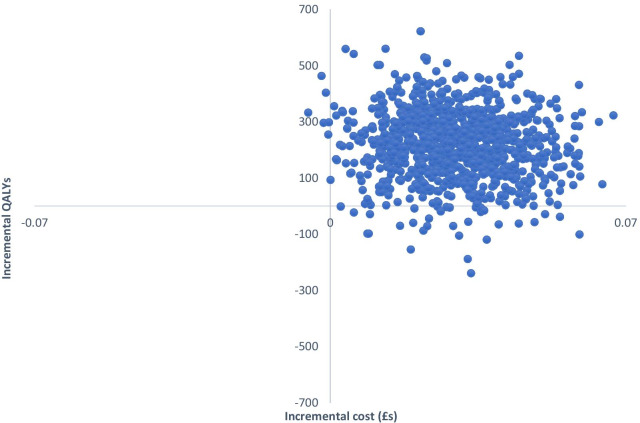
Cost-effectiveness plane for WCBT versus TAU (healthcare perspective, complete case analysis)

**Fig. 3:**
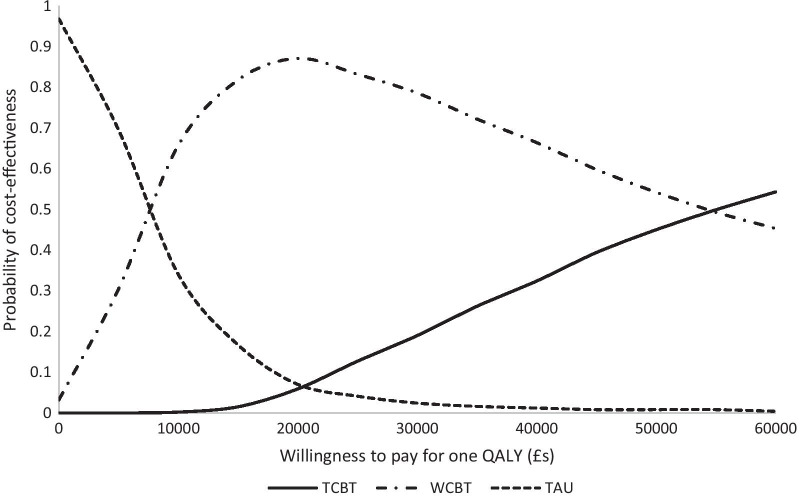
Cost-effectiveness acceptability curves based on QALYs

Uncertainty around the ICERs is illustrated in Figs. 1, 2 and 3. Each point on the cost-effectiveness planes represent a pair of incremental costs and QALYs from 1000 bootstrapped samples. From Fig. 1 we see that TCBT compared to TAU is certain to result in higher healthcare costs and to produce more QALYs. Comparing WCBT to TAU (Fig. 2), there is a 96.2% likelihood that WCBT has higher healthcare costs than TAU and produces more QALYs, a 3.2% likelihood of lower costs and more QALYs, a 0% likelihood of lower costs and fewer QALYs, and a 0.6% likelihood of higher costs and fewer QALYs. The cost-effectiveness acceptability curves show that at very low values placed on a QALY gain, TAU is likely to be the most cost-effective option (Fig. 3). When higher values are placed on a QALY, WCBT becomes the most likely to be cost-effective. The likelihood that TCBT is the most cost-effective option does increase but only exceeds the probability of WCBT at around £55,000 per QALY. At a £20,000 threshold (commonly used in evaluations in England), WCBT is most likely to be cost-effective, followed by TAU and then TCBT.

### Sensitivity analyses

After multiple imputation for missing EQ-5D-5L-based utility scores and costs it was found that TCBT had an ICER of £27,436 per QALY compared to TAU. WCBT resulted in an ICER of £17,388 per QALY. When therapy costs were reduced by 25% and 50% the ICERs from the healthcare perspective compared to TAU were reduced for both groups and the ICER for TCBT was now well below the £20,000 threshold (Table 6). When therapy costs were increased the WCBT option was still well below the £20,000 threshold.

**Table 6:** Sensitivity analyses based on different therapy costs

	Incremental costs and ICERs (£s) following reductions/increases in therapy costs				
	Therapy 50% less	Therapy 25% less	Base case	Therapy 25% more	Therapy 50% more
TCBT incremental cost	614	785	956	1126	1297
ICER	14,312	18,298	22,284	26,247	30,233
WCBT incremental cost	97	160	224	288	352
ICER	3345	5517	7724	9931	12,138

When the minimum wage was used to value informal care and lost work days, mean societal costs reduced to £2362 for TCBT, £1520 for WCBT and £2518 for TAU. After adjusting for baseline, TCBT had costs that were £45 higher than for TAU (bootstrapped 95% CI, − £1145 to £1124) and WCBT had costs that were £389 lower than for TAU (bootstrapped 95% CI, £391 to £1283). When the cost of a homecare worker was used to value informal care, the mean societal costs were £3281 for TCBT, £2275 for WCBT and £5098 for TAU. After adjusting for baseline, TCBT had costs that were £1228 lower than for TAU (bootstrapped 95% CI, £1061 to £3917) and WCBT had costs that were £1241 lower than for TAU (bootstrapped 95% CI, £498 to £3351).

## Discussion

These cost-effectiveness analyses found that TCBT and WCBT resulted in increased healthcare costs over the follow-up period. Higher costs can be justified if outcomes are improved sufficiently. The complete case analysis revealed that the cost per QALY for WCBT was lower than the threshold often assumed to guide NICE decisions in England (£20,000). TCBT on the other hand had a cost-effectiveness ratio slightly above this threshold. On this basis, WCBT would be the preferred option.

However, there was much missing EQ-5D-5L data. This was due to the requirement placed on the trial to use the EuroQol online website to collect the EQ-5D data which required electronic transfer of participants from the trial data collection software platform to the EQ-5D website to input the data. This did not always work effectively, and so data collection was not always completed. With imputation for the missing data, the ICERs both increased substantially. TCBT would be now more unlikely to be considered cost-effective at such a ratio (the ICER being over £30,000) but WCBT would still be below the NICE threshold.

A strength of the study was the comprehensive approach to costing adopted. Both TCBT and WCBT resulted in lower societal costs than TAU. This was because of reduced informal care from family and friends compared to TAU. There was though no evidence that lost work time differed between the groups. From this societal perspective we may deduce that TCBT and WCBT both dominate TAU (i.e. they are more effective and less expensive). However, the confidence intervals are wide and do not exclude zero cost differences. NICE do not usually consider carer costs in their decision-making process though it could be argued with increasing integration of health and social care services and budgets that these are becoming more important to consider. This is a key strength of the study as interventions such as these seem to have benefits outside of the healthcare system. From an economic perspective all relevant effects should be included and costs computed.

Elsewhere, Andersson et al found that internet-based therapy resulted in reduced societal costs for patients compared to participating in a discussion group. [19]. This coupled with improved outcomes resulted in internet therapy being highly cost-effective. In a subsequent study, Ljótsson et al found similar results when comparing internet therapy to a waiting list condition. In our study we have focussed on healthcare costs given that these are the main consideration for NICE in England [4].

### Limitations

There were limitations to this economic evaluation. First, and most importantly, the quantity of missing EQ-5D-5L data was a concern. Those with missing data had worse IBS-SSS and WASAS scores at each time point and so imputation from these resulted in smaller QALY gains for TCBT and WCBT relative to TAU (details available from authors).

Second, service use data were provided by participants themselves and there may have been recall accuracy problems. However, this would not likely affect one group more than another and it was the only option for collecting comprehensive data. Third, medication data were not of high quality. We did know what medications were taken but quantities and durations were not complete. Many medications were bought ‘over the counter’ and exact details may not have always been available. However, these costs would be a small proportion of the total healthcare cost and their inclusion would have only a marginal effect. We might though have expected TAU to result in higher medication costs in the absence of an active therapy.

Third, the economic analyses were trial based and so limited to the 12-month follow-up period. It could well be that economic impacts continue beyond this time and modelling to extrapolate these results is warranted.

Finally, the intervention costs for WCBT were apportioned over the trial population. If both forms of ACTIB CBT were rolled out over a larger patient population then the fixed costs of the intervention would fall per patient.

We believe this to be the largest trial of CBT for IBS worldwide to date and the first to evaluate telephone and web-based CBT in the same trial. It was a rigorously conducted trial with broad inclusion criteria and patients recruited from both primary and secondary care. The trial interventions were provided by NHS therapists in a real-world NHS setting. The inclusion of the cost-effectiveness component provides further important evidence for policy makers.

## Conclusions

In conclusion, the complete case analysis suggests that both the therapies are cost-effective from a healthcare perspective. This is reduced when using imputation methods, but the web-based CBT intervention remained cost-effective. Both interventions produce important savings in terms of carer time and if this is valued then the interventions do cover their cost. These results add important research evidence to support recommendations in NICE Guidelines that CBT should be offered to people with ongoing IBS symptoms.

## Data Availability

PMc can be contacted at the corresponding email address regarding data-sharing requests, including access to the patient self-management, therapist training, and therapist manuals. Individual participant data that underlie the results reported in this Article might be available after de-identification to researchers who provide a methodologically sound proposal and whose proposed use of the data has been approved by an independent review committee. To gain access, data requesters will need to sign a data access agreement.

## Abbreviations

CBT: Cognitive behavioural therapy
CEAC: Cost-effectiveness acceptability curve
CEP: Cost-effectiveness plane
CSRI: Client Service Receipt Inventory
IBS: Irritable bowel syndrome
NICE: National Institute for Health and Care Excellence
QALY: Quality-adjusted life year
TAU: Treatment as usual
TCBT: Telephone-delivered cognitive behavioural therapy
WCBT: Web-based cognitive behavioural therapy
